# Distinct early development trajectories in *Nf1*^±^ and *Tsc2*^±^ mouse models of autism

**DOI:** 10.1186/s11689-025-09624-6

**Published:** 2025-07-26

**Authors:** Helena Ferreira, Sofia Santos, João Martins, Miguel Castelo-Branco, Joana Gonçalves

**Affiliations:** 1 University of Coimbra, Coimbra Institute for Biomedical Imaging and Translational Research (CIBIT), Health Sciences Campus, Azinhaga de Santa Comba, Celas, Coimbra, 3000-548 Portugal; 2 University of Coimbra, Institute of Nuclear Sciences Applied to Health (ICNAS), Coimbra, Portugal; 3 University of Coimbra, Institute of Physiology, Faculty of Medicine, Coimbra, Portugal

**Keywords:** ASD, Neurofibromatosis type 1, Tuberous sclerosis complex 2, Developmental milestones, Ultrasonic vocalizations

## Abstract

**Background:**

Autism spectrum disorder (ASD) is a neurodevelopmental condition characterized by deficits in social communication and interaction, and repetitive behaviors. Males are three times more likely to be diagnosed with ASD than females, and sex-dependent alterations in behavior and communication have been reported both in clinical and animal research. Animal models are useful for understanding ASD-related manifestations and their associated neurobiological mechanisms. However, even though ASD is diagnosed during childhood, relatively few animal studies have focused on neonatal development.

**Methods:**

Here, we performed a detailed analysis of neonatal developmental milestones and maternal separation-induced ultrasonic vocalizations (USVs) in two genetic animal models of ASD, neurofibromatosis type 1 (*Nf1*^±^) and tuberous sclerosis complex 2 (*Tsc2*^±^).

**Results:**

*Nf1*^±^ and *Tsc2*^±^ mice display strikingly distinct developmental profiles regarding motor, strength, and coordination skills. *Nf1*^±^ mouse pups mostly show genotype-related differences, whereas *Tsc2*^±^ mouse pups mainly present sexual dimorphisms. Furthermore, we found several differences regarding the number of USVs, frequency modulation, and temporal and spectral profile. Importantly, *Nf1*^±^ animals tend to present sex- and genotype-dependent differences earlier than the *Tsc2*^±^ mouse pups, suggesting distinct developmental curves between these two animal models.

**Conclusions:**

This study provides a nuanced understanding of how these two ASD models differ in their developmental trajectories. It underscores the importance of studying sex differences and early-life developmental markers, as these could offer crucial insights into ASD's progression and neurobiology. The distinct profiles of these models may help guide more targeted therapeutic strategies in the future.

**Supplementary Information:**

The online version contains supplementary material available at 10.1186/s11689-025-09624-6.

## Background

Autism spectrum disorder (ASD) is a neurodevelopmental condition affecting 1 in every 36 children [1]. It is characterized by deficits in social communication and interaction, and restrictive, repetitive patterns of behavior or activities [2]. Diagnosis of ASD can start being accurately performed only in 2–3-year-old children, through observation of issues in social activities, impaired communication, and abnormal behaviors [3]. This disorder is marked by a great heterogeneity in etiology, where genetic, epigenetic, and environmental factors may contribute to the pathogenetic pathways increasing vulnerability to ASD [4].

While no animal model captures the full complexity of ASD symptoms in humans, models based on ASD-causing mutations are quite useful for studying the neurobiological basis of ASD-related behaviors. Our group and others have studied two well-established animal models of genetic ASD, neurofibromatosis type 1 (*Nf1*^±^) and tuberous sclerosis complex 2 (*Tsc2*^±^) [5–8]. Interestingly, while *Nf1*^±^ is characterized by increased inhibitory tone, *Tsc2*^±^ has an excitatory signature; this allows us to reach the overall spectrum of autism spectrum’s etiological heterogeneity [7–9].

Furthermore, impaired communication is a hallmark of ASD that also requires attention. Indeed, ASD children display difficulties in placing adequate stress, pitch, loudness, and resonance in their speech [10]. Further, diminished vocalization production, reduced canonical syllables, and increased non-syllabic or atypical vocalizations in their speech have been reported [11–13]. Importantly, early vocalization acoustics and language skills of infants can be used as a behavioral biomarker for a later diagnosis of ASD [14], underscoring the need for a detailed study of early vocalizations in ASD. Ultrasonic vocalizations (USVs), along with olfaction, are a key aspect in mouse communication [15], with infant mice mainly producing USVs when isolated, to elicit retrieval by their dam [16]. USVs have been shown to provide insight into the emotional and developmental state of the animal [17–24], making USVs extremely useful in the study of behavior and developmental disorders.

Finally, growing evidence demonstrates variations in physiology and regulation of neural circuits between male and female subjects, which directly reflect on different susceptibilities to mental disorders [25]. ASD is no exception, with male individuals being three times more likely to present the disease than females [26], with a consequential greater investigation of ASD in male subjects [27]. However, in recent years, the scientific community has agreed on the necessity to increase the research of ASD on females and unravel sexual dimorphisms in its manifestations and underlying mechanisms. Sex-dependent and sex-specific alterations regarding behavior, communication, and cognitive ability have been reported, both in human and animal studies [27–32].

Given the heterogeneity in symptoms and etiology of this disorder, it is important to understand how different mouse models of ASD differ during development at the perinatal period, concerning their early behavior and communication. Further, as biological sex has shown to be a significant factor in the manifestation of ASD, it is crucial to investigate both male and female subjects.

## Methods

Here, we studied the behavioral development and communication skills during the infancy of male and female *Nf1*^±^ and *Tsc2*^±^ mice.

### Animals

*Nf1*^±^ mice and their WT^*Nf1*^ littermates were obtained from mutant animals C57BL/6N backcrossed only once to 129T2/SvEmsJ as previously reported [33, 34]. *Tsc2*^±^ mice and their WT^*Tsc2*^ littermates were generated from backcrossing C57BL/6 J to C57BL/6N animals [7, 35]. In all breeding schemes, the female was WT and the male was mutant, to eliminate possible confounding effects of maternal behavior in the litters’ behavior and development. 12 *Nf1* litters, with 4–11 pups per litter, and 10 *Tsc2* litters, with 6–9 pups per litter, were used in this study. Both animal strains were generated and maintained in our animal facilities at ICNAS (University of Coimbra), in a housing room with a 12 h/12 h light–dark cycle, at 21 ± 2 °C. *Nf1* and *Tsc2* animal models were used during the infancy period, from postnatal day (PND) 6 to PND10. At PND3, pups were identified with permanent tattoos on their toes, and tails were collected for genotyping. All pups were housed together with the dam until PND21, at which point each litter was segregated by sex. Developmental milestone tests and USV recordings were performed at PND6, PND8 and PND10, in the same sequence across the entire experiment, during the light period (8:00 AM – 11:00 AM). At the end of the battery of tests, each pup was weighed, measured, and returned to its home cage. The operator was blind to genotype and sex. All experiments are following the European Union Council Directive (2010/63/EU), National Regulations, and ORBEA board of ICNAS (1/2017).

### Developmental behavioral testing

Developmental behaviors were assessed on PND6, PND8 and PND10, with each specific behavior being analyzed in the indicated age range. On PND6 and PND8, developmental behavior tests were performed in the following order: surface righting reflex test, followed by negative geotaxis test. On PND10, developmental behavior tests were performed in the following order: locomotion test, followed by nest-seeking test. Between each test on each day, the animals were allowed to rest for 1 min.

### Surface righting reflex

To assess motor skills and the development of the vestibular system, on PND6 and PND8, the pup was placed on its back on a flat surface (laboratory bench covered with one layer of tissue paper) and was held in that position by the operator for 5 s. The time taken by the animal to return to a four-limb position after release was registered, with a cut-off time of 30 s [36] (Additional File 1 A).

### Negative geotaxis reflex

Again to investigate the developmental stage of motor skills and of the vestibular system of the animals, on PND6 and PND8, a ramp with a 35° inclination, covered with fabric to allow traction, was prepared. The animal was placed facing down on the apparatus, and the time taken to rotate and orient its forepaws to the top was measured, with a cut-off time of 30 s. In case the animal fell or rolled down the platform, it was given 2 extra tries maximum, after which a score of 30 s was attributed [36] (Additional File 1B).

### Locomotion

To assess spontaneous locomotor activity and development of motor skills, on PND10, the animal was placed in the center of a 13 cm diameter circle, drawn on a paper towel fixed on the surface of a laboratory bench. The time the animal took to cross the limit of the circle with both forepaws was measured, with a cut-off time of 30 s [36] (Additional File 1 C).

### Nest-seeking behavior

To test the development of the sensory system, namely, olfactory discrimination skills, on PND10, a rectangular plastic arena (25 cm × 10 cm) was divided into 3 compartments. Home bedding was placed on the left compartment, and fresh bedding in a similar amount was placed on the right compartment. A goal line was traced in each compartment 6,5 cm from the center. The animal was placed in the middle compartment, at a 90° angle from the bedding compartments, and allowed to explore the arena. Two trials were performed, with a 30-s intertrial between them, during which the operator held the animal. In each trial, latency to cross the home bedding goal line with both forepaws was measured, with a cut-off time of 120 s. If the animal did not perform or crossed the fresh bedding goal line with both forepaws, the cut-off score was attributed. The final score was calculated by averaging the scores attributed to each trial. In each trial, the animal was positioned facing opposite sides of the arena to even out possible side-turning preferences. During the experiment with all the animals, the first trial and second trial positions of the animals in the arena were alternated [36] (Additional File 1D).

### Ultrasonic vocalization recordings and analysis

A 55 cm × 50 cm × 70 cm (H x D x W) anechoic chamber was assembled with 1,5 cm thick acrylic sheets, and fully covered with absorbing foam on the inside, to block external sound. All USVs were acquired using an ultrasound recording system with Avisoft CM16/CMPA condenser microphone, UltrasoundGate 416H amplifier, and Avisoft Recorder software (Avisoft Bioacoustics, Glienicke/Nordbahn, Germany). Pup USV recording occurred on PND6, 8 and 10 during the light period (8:00 AM – 11:00 AM). Each pup was removed from the home cage (maternal separation) and placed into a 15 × 10 × 8 plastic container, padded with tissue paper to help maintain body temperature. The pup was acclimated to the box for 2 min, after which the sound recording started with the condenser microphone placed 28 cm above the container's bottom and lasted 5 min. At the end of the test, the pup was returned to its home cage. Sonograms were generated, with FFT-length 512 points, 16-bit format, sampling frequency 250 kHz, time resolution 1 ms, frequency resolution 488 Hz, overlap 50%. USVs were further analyzed using MATLAB toolbox DeepSqueak version 2.6.1., which allowed the extraction of individual mouse USV calls by applying the Mouse Call_Network_V2 neural network, with a chunk length analysis of 6 s, overlap of 0.1 s, high frequency cut-off of 125 kHz and no score threshold. Each USV was manually classified into three classes, similarly to the work of Young et al. (2010) [37]. USV duration, total USV time, latency to first USV, inter-USV interval duration, mean frequency and slope (frequency variation over time) were also analyzed.

### Statistical analyses

All data was analyzed using GraphPad Prism version 8.0.1 (GraphPad Software, California, USA). Outliers were identified as values outside the mean ± 3SD interval and were excluded. One- or two-way analysis of variance (ANOVA) with age as repeated measure, followed by Tukey’s post-hoc test, with correction for multiple comparisons, was used to compare experimental groups. Both genotype and sex were included as between-subject factors in the analysis. *Nf1* (*Nf1*^±^ and WT^*Nf1*^) and *Tsc2* (*Tsc2*^±^ and WT^*Tsc2*^) litters were analyzed separately. All effects are reported as significant at *p* < 0.05. Error bars are given as SEM.

## Results

### Transgenic animals have sex-induced changes in body weight and length in early days of life

WT and transgenic animals were weighted along infancy period at PND6, PND8 and PND10, immediately after developmental milestones tests. We found that in both *Nf1*^±^ and *Tsc2*^±^ mice there was a significant overall effect of genotype in weight [*Nf1*^±^: F(3,228) = 3.187, *p* = 0.0246; *Tsc2*^±^: F(3,230) = 5.779, *p* = 0.0008] and in length [*Nf1*^±^: F(3,228) = 5.166, *p* = 0.0018; *Tsc2*^±^: F(3,236) = 6.930, *p* = 0.0002]. Age also produced a significant overall effect in weight [*Nf1*^±^: F(2,228) = 111.1, *p* < 0.0001; *Tsc2*^±^: F(2,230) = 107.1, *p* < 0.0001] and length [*Nf1*^±^: F(2,228) = 193.1, *p* < 0.0001; *Tsc2*^±^: F(2,236) = 116.7, *p* < 0.0001] of all experimental groups used in this study, as expected during this age of fast development. Although post-hoc analysis revealed that no differences were found in weight between any *Nf1*^±^ experimental groups (Additional File 2 A), changes in length occurred only in PND6, fading as the pups grew, in a sex-dependent or -specific manner. Specifically, Tukey’s multiple comparisons test showed that 6-day-old male *Nf1*^±^ pups were longer than transgenic females at the same age (Additional File 2B, Additional File 3). Concerning *Tsc2*^±^ animals, a similar trend is found with the WT^*Tsc2*^ littermates, as males had a greater length than females (Additional File 2D, Additional File 4). Finally, female *Tsc2*^±^ mice were heavier than WT^*Tsc2*^ female littermates (Additional File 2 C, Additional File 4).

### Both Nf1^±^ and Tsc2^±^ mutations lead to neonatal reflex and behavior alterations

**Fig. 1:**
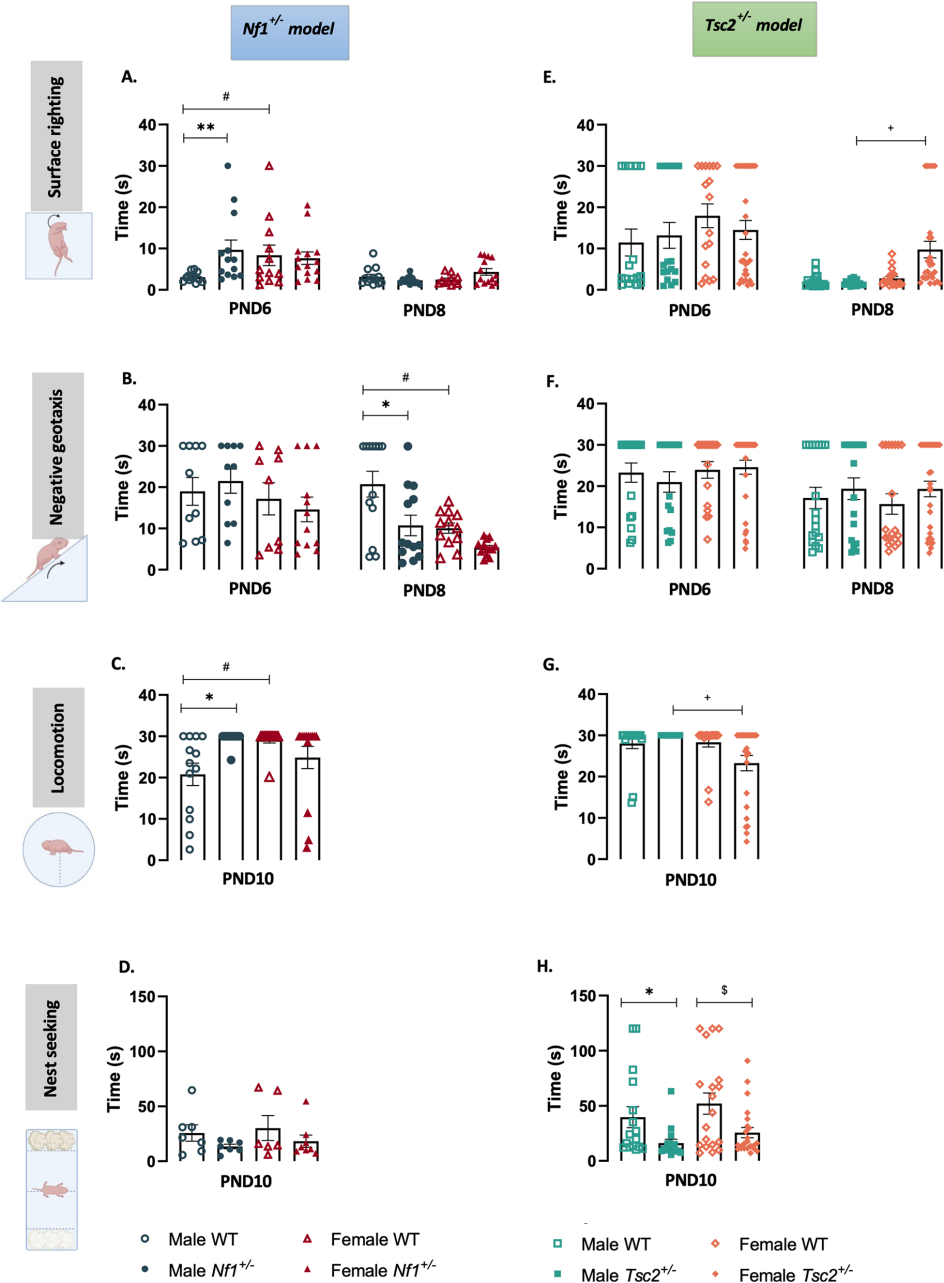
Developmental milestones. Surface righting, negative geotaxis, locomotion and nest seeking tests were performed in male and female mouse pups of *Nf1*^±^ (**A**, **B**, **C** and **D**, respectively) and *Tsc2*^±^ (E, F, G and H, respectively) experimental groups from PND6 until PND10. Two-way ANOVA (surface righting and negative geotaxis tests) or one-way ANOVA (locomotion and nest seeking tests), followed by Tukey’s multiple comparisons test. Data represented as mean ± SEM; n (male WT^*Nf1*^) = 18–19; n (female WT^*Nf1*^) = 19–20; n (male *Nf1*^±^) = 18–19; n (female *Nf1*^±^) = 20–22; n (male WT^*Tsc2*^) = 16–18; n (female WT^*Tsc2*^) = 16–20; n (male *Tsc2*^+*/*^) = 16–19; n (female *Tsc2*.^±^) = 25–30

To investigate whether *Nf1*^±^ and *Tsc2*^±^ mutations impacted neonatal behavior and development, four different neurobehavioral tests, namely surface righting reflex, negative geotaxis reflex, locomotion (extinction of pivoting and crawling) and nest-seeking behavior tests were performed (Fig. 1, Additional Files 5 and 6).

Regarding the surface righting test, it was found an overall effect of age [F(1,95) = 18.05, *p* < 0.0001], as well as an interaction between genotype and age in the *Nf1*^±^ mouse model [F(3,95) = 2.834, *p* = 0.0424]. Further, we found that both *Nf1*^±^ males and WT^*Nf1*^ females spent more time reaching the upright position than WT^*Nf1*^ males at PND6, without significant differences at PND8 (Fig. 1A, Additional File 5). On the contrary, in *Tsc2*^±^ animals, there was an effect of genotype [F(3,149) = 3.017, *p* = 0.0319] and of age [F(1,149) = 38.96, *p* < 0.0001], with no interaction effect. Multiple comparison tests showed that no changes between the groups were detected in PND6. However, at PND8 transgenic females took longer to upright themselves than *Tsc2*^±^ males (Fig. 1E, Additional File 6).

In the negative geotaxis reflex of the *Nf1*^±^ animal model, again, we found an effect of genotype [F(3,95) = 5.324, *p* = 0.0020] and of age [F(1,95) = 11.00, *p* = 0.0013], as well as an interaction between these two factors [F(3,95) = 3.218, *p* = 0.0263]. Interestingly, it was observed that male *Nf1*^±^ and female WT^*Nf1*^ mice decreased the head-turning time in geotaxis reflex compared with male WT^*Nf1*^ littermates on PND8 (Fig. 1B, Additional File 5). No changes were found in the *Tsc2*^±^ animal model regarding performance in the negative geotaxis test, with only an effect of age being found [F(1,160) = 10.82, *p* = 0.0012] (Fig. 1F, Additional File 6).

Regarding locomotory development, both ASD models showed sex differences [*Nf1*^±^ model: F(3,48) = 4.069, *p* = 0.0118; *Tsc2*^±^ model: F(3,71) = 4.324, *p* = 0.0074]. However, strikingly, while it is the male *Nf1*^±^ and WT^*Nf1*^ females that show a decreased development of locomotory skills compared to male WT^*Nf1*^ littermates (Fig. 1C, Additional File 5), in the *Tsc2*^±^ groups it is the mutant females that take less time to acquire adequate locomotor movements, compared to mutant males (Fig. 1G, Additional File 6).

Finally, we performed a nest-seeking task and found significant differences only in the *Tsc2*^±^ animal model [F(3,70) = 5.330, *p* = 0.0023]. Surprisingly, both male and female mutant mice performed significantly better in the nest-seeking task, needing less time to reach home bedding (Fig. 1H, Additional File 6).

### Features of USVs of Nf1^±^ and Tsc2^±^ mice are modified by sex and age

**Fig. 2:**
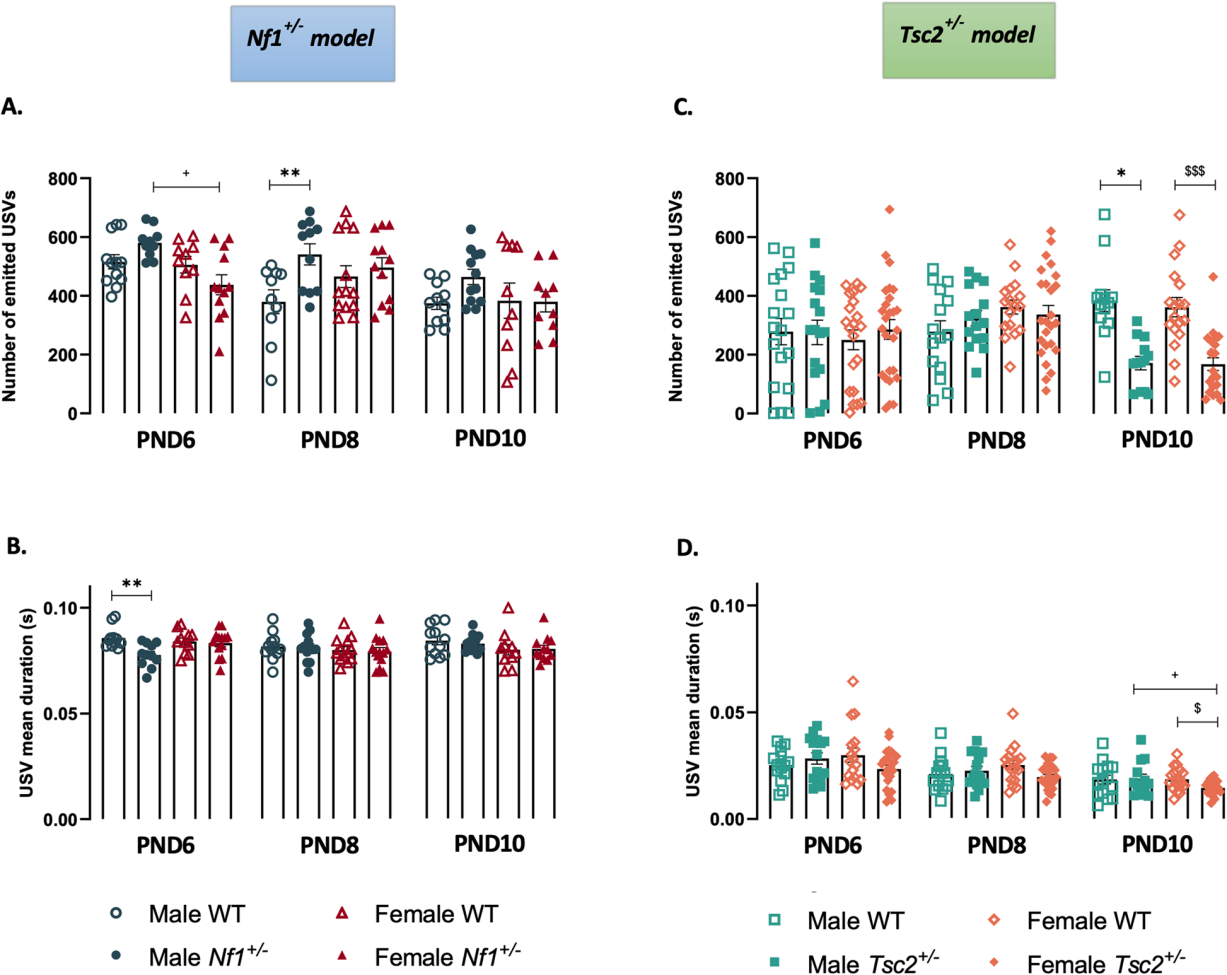
Maternal separation-induced ultrasonic vocalizations I – main features. USVs of male and female mouse pups of *Nf1*^±^ and *Tsc2*^±^ experimental groups were recorded from PND6 until PND10. Total number of USVs (**A** and **C**, respectively) and USV duration (**B** and **D**, respectively) were analyzed. Two-way ANOVA followed by Tukey’s multiple comparisons test. Data represented as mean ± SEM; n (male WT^*Nf1*^) = 18–19; n (female WT^*Nf1*^) = 19–20; n (male *Nf1*^±^) = 18–19; n (female *Nf1*^±^) = 20–22; n (male WT^*Tsc2*^) = 16–18; n (female WT^*Tsc2*^) = 16–20; n (male *Tsc2*^±^) = 16–19; n (female *Tsc2*.^±^) = 25–30

To explore early social communication, we recorded and characterized maternal separation-induced USVs of pups at PND6, PND8, and PND10. Our data revealed that the total number of USVs had an overall effect of age on the *Nf1*^±^ mouse model [F(2,117) = 4.509, *p* = 0.0130]. However, following the analysis with post-hoc multiple comparisons tests, we found that at PND6 *Nf1*^±^ males displayed a greater number of emitted USVs than transgenic females. Moreover, transgenic males vocalized more than WT^*Nf1*^ male mice, however, this comparison only reached statistical significance at PND8 (Fig. 2A, Additional File 7). Interestingly, however, it was at this time point that the difference in USV duration between the WT^*Nf1*^ and transgenic males disappeared, as *Nf1*^±^ males produced significantly shorter USVs than WT^*Nf1*^ males on PND6 (Fig. 2B, Additional File 7).

Contrary to *Nf1*^±^ animals, changes in the *Tsc2*^±^ mouse model were only found at PND10 (Fig. 2C and D, Additional File 8). Indeed, concerning *Tsc2*^±^ animals, we found an overall effect of genotype [F(3,196) = 5.261, *p* = 0.0016], age [F(2,196) = 32.90, *p* < 0.0001], and an interaction between these two factors [F(6,196) = 2.599, *p* = 0.0191] on the number of produced USVs. Specifically, total number of emitted USVs showed a decrease in mutant animals of both sexes (Fig. 2C, Additional File 8). Additionally, there was an effect of genotype [F(3,214) = 3.355, *p* = 0.0198] and age [F(2,214) = 16.06, *p* < 0.0001] on USV duration. Interestingly, the *Tsc2*^±^ females presented the shortest calls compared with both transgenic males and WT^*Tsc2*^ females (Fig. 2D, Additional File 8).

**Fig. 3:**
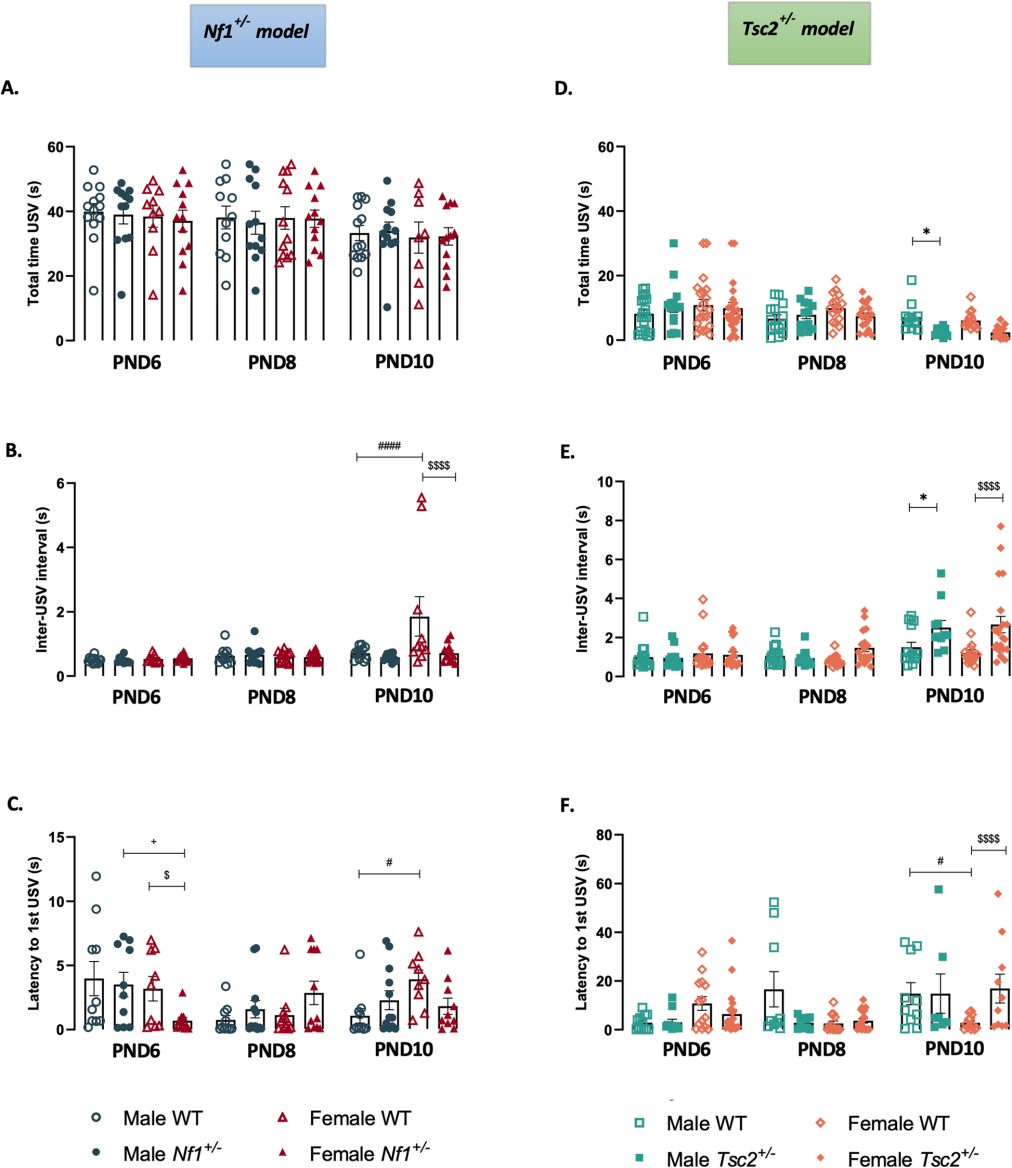
Maternal separation-induced ultrasonic vocalizations II – temporal profile. Total time emitting USVs (**A** and **D**), inter-USV interval (**B** and **E**) and latency to produce first USV (C and F) of male and female mouse pups of *Nf1*^±^ and *Tsc2*^±^ experimental groups, respectively, from PND6 until PND10. Two-way ANOVA followed by Tukey’s multiple comparisons test. Data represented as mean ± SEM; n (male WT^*Nf1*^) = 18–19; n (female WT^*Nf1*^) = 19–20; n (male *Nf1*^±^) = 18–19; n (female *Nf1*^±^) = 20–22; n (male WT^*Tsc2*^) = 16–18; n (female WT^*Tsc2*^) = 16–20; n (male *Tsc2*^±^) = 16–19; n (female *Tsc2*.^±^) = 25–30

Notably, when we analyzed the total time during which the animals emitted USVs we found differences between the mouse models used in this work (Fig. 3A and D, Additional Files 9 and 10). While in *Nf1*^±^ mice there was only an overall effect of age [F(2,127) = 3.820, *p* = 0.0245] with no differences between groups in the total time used to emit vocalizations (Fig. 3A, Additional File 9), in the *Tsc2*^±^ animal model two-way ANOVA showed an effect of genotype [F(3,168) = 4.083, *p* = 0.0079] and of age [F(2,168) = 17.77, *p* < 0.0001]. With a multiple comparisons test, we found that male *Tsc2*^±^ mice spent less total time vocalizing than their WT^*Tsc2*^ littermates at PND10 (Fig. 3D, Additional File 10).

Looking for inter-USV interval, which represents the mean silence time mediating the end of one USV and the beginning of the next emitted USV, we detected differences in both *Nf1*^±^ and *Tsc2*^±^ animals at PND10 (Fig. 3B and E). Indeed, an overall effect of age [*Nf1*^±^: F(2,124) = 9.224, *p* = 0.0002; *Tsc2*^±^: F(2,178) = 18.22, *p* < 0.0001], genotype [*Nf1*^±^: F(3,124) = 3.998, *p* = 0.0093; *Tsc2*^±^: F(3,178) = 5.446, *p* = 0.0013], and an interaction between the them [*Nf1*^±^: F(6,124) = 4.189, *p* = 0.0007; *Tsc2*^±^: F(6,178) = 3.024, *p* = 0.0077] was observed. Interestingly, female WT^*Nf1*^ littermates showed the longest inter-USV interval (Fig. 3B, Additional File 9), and again, male and female *Tsc2*^±^ mice presented a significant increase in time between calls compared with their WT^*Tsc2*^ littermates (Fig. 3E, Additional File 10).

Finally, we also report alterations in the latency time to the first USV emission (Fig. 3C and F, Additional Files 9 and 10). It was found an overall effect of age [F(2,119) = 3.671, *p* = 0.0284], and a genotype x age interaction [F(6,119) = 3.981, *p* = 0.0011]. *Nf1*^±^ females were the experimental group that took less time to emit a USV after maternal separation at PND6, however, at PND10, we only observed a significant difference between WT^*Nf1*^ males and females (Fig. 3C). Concerning *Tsc2*^±^ mice, once more, there was an overall effect of age [F(2,168) = 9.432, *p* = 0.0001], genotype [F(3,168) = 2.784, *p* = 0.0425], and their interaction [F(6,168) = 5.185, *p* < 0.0001], regarding latency to first USV emission. WT^*Tsc2*^ males and transgenic females took more time to start vocalizing after the beginning of the test at PND10, compared to female WT^*Tsc2*^ mice (Fig. 3F, Additional File 10). This parameter in particular seems to show great variation over time, which limits the ability to draw firm conclusions from this data.

It is interesting to note that the *Tsc2*^±^ and WT^*Tsc2*^ experimental groups consistently showed genotype- and sex-dependent differences in their USVs on PND10 specifically, while the *Nf1*^±^ and WT^*Nf1*^ groups started to show dimorphisms earlier in time.

**Fig. 4:**
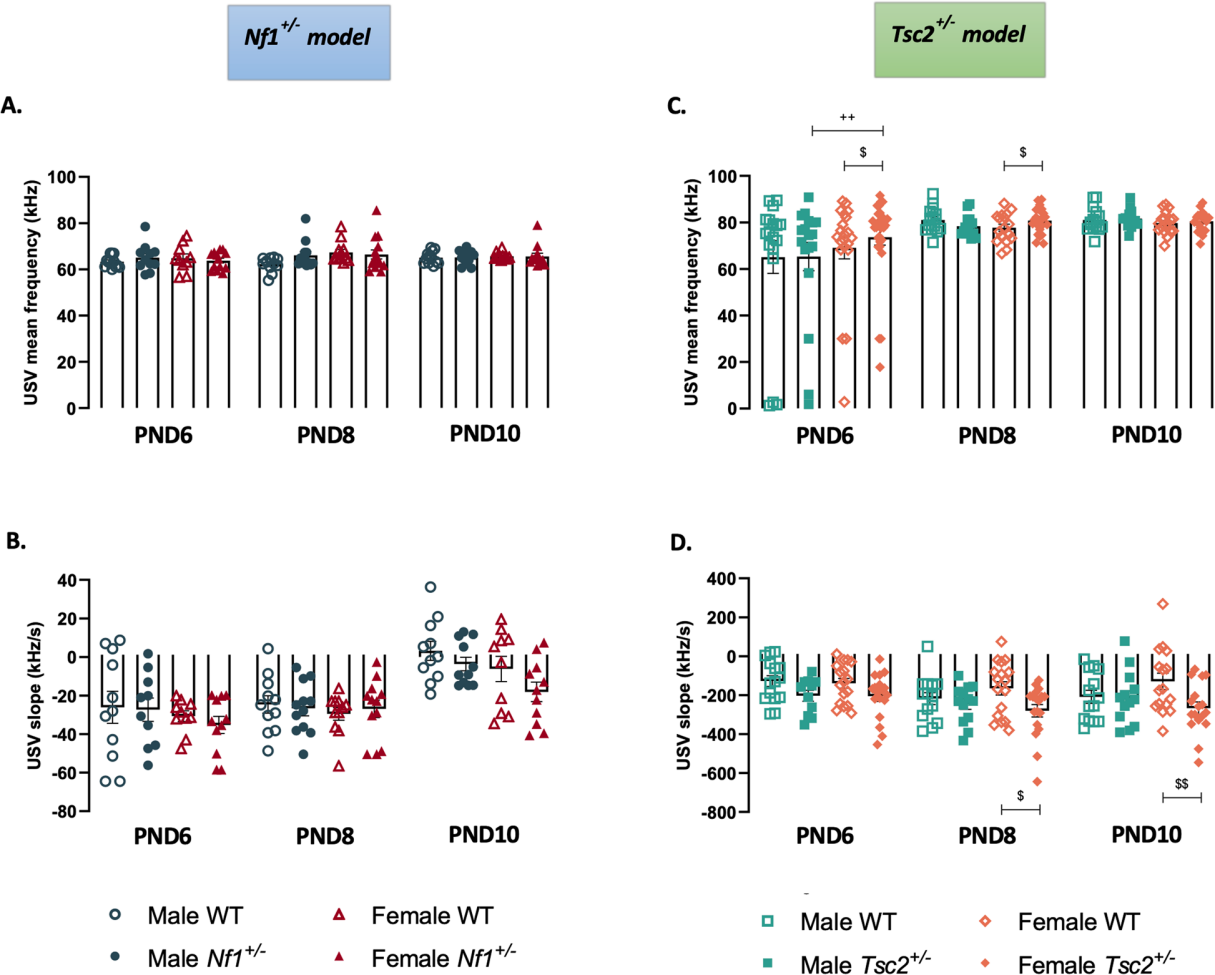
Maternal separation-induced ultrasonic vocalizations III – frequency profile. Frequency (**A** and **C**) and slope (**B** and **D**) of male and female mouse pups of *Nf1*^±^ and *Tsc2*^±^ experimental groups, respectively, from PND6 until PND10. Two-way ANOVA followed by Tukey’s multiple comparisons test. Data represented as mean ± SEM; n (male WT^*Nf1*^) = 18–19; n (female WT^*Nf1*^) = 19–20; n (male *Nf1*^±^) = 18–19; n (female *Nf1*^±^) = 20–22; n (male WT^*Tsc2*^) = 16–18; n (female WT^*Tsc2*^) = 16–20; n (male *Tsc2*^±^) = 16–19; n (female *Tsc2*.^±^) = 25–30

To better characterize USVs, average frequency and slope (frequency variation over time) were investigated (Fig. 4, Additional Files 11 and 12). Only an effect of age [F(2,119) = 26.01, *p* < 0.0001] was found in the USV slope of the *Nf1*^±^ mouse model, with no changes between the groups in any of the parameters (Fig. 4A and B, Additional File 11). On the other hand, in *Tsc2*^±^ animals both parameters showed alterations, with an effect of age [USV frequency: F(2,218) = 10.33, *p* < 0.0001] and genotype [USV frequency: F[3,218) = 4.782, *p* = 0.0030; USV slope: F(3,171) = 7.312, *p* = 0.0001]. In fact, at PND6, transgenic females presented the higher mean frequency, which continues higher than WT^*Tsc2*^ female littermates at PND8 (Fig. 4C, Additional File 12). Additionally, differences between *Tsc2*^±^ females and their WT^*Tsc2*^ female littermates were also present in USV slope at PND8 and PND10 (Fig. 4D), with a higher absolute value in transgenic females.

### USV syllables are modified in a sex- and genotype-dependent manner

It has been established that the mouse USV repertoire contains different types of USV calls, or syllables [38, 39]. Here, we categorized pup vocalizations based on their temporal and frequency properties, by identifying single calls, containing a single component, multisyllabic calls, containing consecutive components which are separated in time, and stacked calls, where simultaneously emitted components are separated in frequency space [37].

**Fig. 5:**
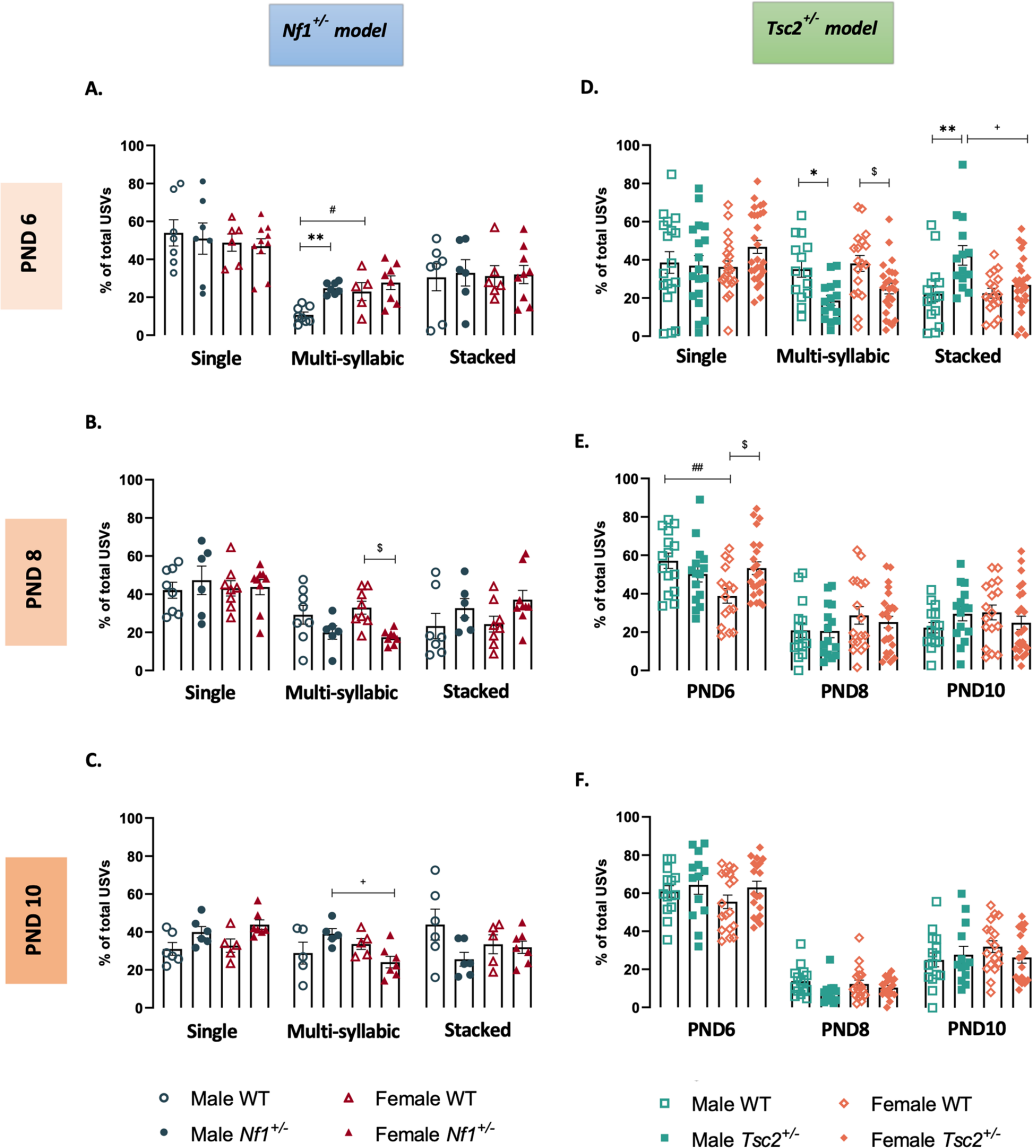
Maternal separation-induced ultrasonic vocalizations IV – syllable composition. Percentage of single, multisyllabic and stacked syllables, on PND6, PND8 and PND10, of the USVs of male and female mouse pups of *Nf1*^±^ (**A**, **B** and **C**) and *Tsc2*^±^ (**D**, **E** and **F**) experimental groups. One-way ANOVA followed by Tukey’s multiple comparisons test. Data represented as mean ± SEM; n (male WT^*Nf1*^) = 18–19; n (female WT^*Nf1*^) = 19–20; n (male *Nf1*^+*/*^) = 18–19; n (female *Nf1*^±^) = 20–22; n (male WT^*Tsc2*^) = 16–18; n (female WT^*Tsc2*^) = 16–20; n (male *Tsc2*^±^) = 16–19; n (female *Tsc2*.^±^) = 25–30

In the *Nf1*^±^ mouse model, we observed differences between groups in multisyllabic calls across all ages [PND6: F(3,24) = 7.692, *p* = 0.0009; PND8: F(3,26) = 4.486, *p* = 0.0115; PND10: F(3,34) = 3.324, *p* = 0.0311]. Indeed, at PND6, *Nf1*^±^ males and WT^*Nf1*^ females presented the highest percentage of multisyllabic calls, compared with male WT^*Nf1*^ littermates (Fig. 5A, Additional File 13), showing a sexual dimorphism between WT^*Nf1*^ animals that is not found in transgenic animals. Interestingly, this phenotype seems to be reversed from PND8 onwards, as transgenic females showed a reduction in the percentage of multisyllabic calls at PND8 (Fig. 5B, Additional File 13) and PND10 (Fig. 5C, Additional File 13), here again reflecting a sexual dimorphism.

Finally, the syllables produced by the *Tsc2*^±^ animal model showed an overall group effect on PND6 [Multisyllabic USVs: F(3,68) = 6.233, *p* = 0.0008; Stacked USVs: F(3,67) = 5.650; *p* = 0.0016] and on PND8 [Single USVs: F(3,65) = 4.089, *p* = 0.0101]. Moreover, *Tsc2*^±^ animals exhibited a decrease in multisyllabic calls emitted on PND6 by both transgenic males and females, when compared with their WT^*Tsc2*^ littermates. Also, at this age, *Tsc2*^±^ males displayed the higher percentage of stacked USVs, revealing a sexual dimorphism between transgenic animals (Fig. 5D, Additional File 14). Although at PND10 no changes were found between groups (Fig. 5F, Additional File 14), at PND8 we found a sexual dimorphism between WT^*Tsc2*^ males and females in single calls, which was lost in transgenic animals. Indeed, *Tsc2*^±^ females displayed an increase in the percentage of these calls compared to their WT^*Tsc2*^ littermates (Fig. 5E, Additional File 14).

## Discussion

In the present work, we performed a detailed analysis of the developmental profiles of two mouse models of ASD and their WT littermates, regarding coordination, motor and sensory ability, as well as vocalization skills, during the infant age. Although ASDs, including in NF1 and TSC2 individuals, have by definition childhood onset, many of the published works use adult animal models, and relatively little is known about developmental milestone impairments.

**Fig. 6:**
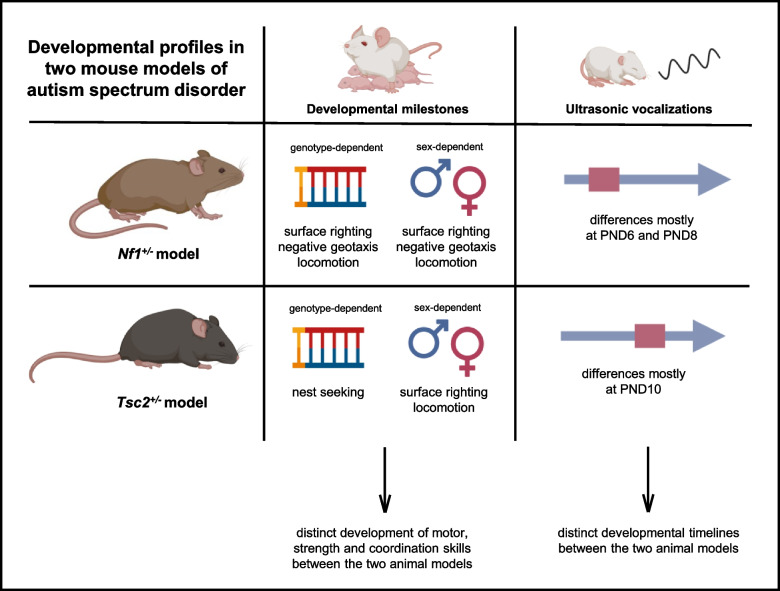
Overview of early development of *Nf1*^±^ and *Tsc2*^±^ mouse pups. The two animal models of autism spectrum disorder revealed differing genotype- and sex-mediated differences in early development, drawing distinct outlines of development over time. *Nf1*^±^ mouse pups showed both sex- and genotype-dependent differences in performance in surface righting, negative geotaxis and locomotion milestones tests; while *Tsc2*^±^ mouse pups displayed sex-driven differences in surface righting and locomotion, and genotype-driven differences in nest seeking skills. Additionally, regarding ultrasonic vocalization production, we found that differences among *Nf1*^±^ mice occurred earlier than in *Tsc2*.^±^ mice, underscoring the very distinct profiles of development shown in these two animal models (created in Biorender.com)

Here, we found that the two used animal models of ASD follow strikingly different developmental outlines during their first days of life. In fact, while *Nf1*^±^ mice showed both abundant genotype- and sex-dependent differences, namely in surface righting, negative geotaxis, and locomotion, *Tsc2*^±^ animals presented a genotype-dependence in their nest-seeking test outcomes. In addition, *Tsc2*^±^ animals also display sex-related dimorphisms between transgenic animals in surface righting and locomotion performances. Additionally, the anatomical analysis revealed that NF1 transgenic animals showed sexual dimorphism in body length, while *Tsc2*^±^ animals displayed both sex- and genotype- dependent body weights and lengths. Overall, we can conclude that both animal models present sexual and genotype-driven differences, in different degrees and different early life developmental tests, as represented in the summary scheme of Fig. 6. This highlights the necessity to thoroughly characterize the several animal models of ASD.

Contrary to our results, Maloney et al. (2018) [30] reports no alterations in developmental milestones in both *Nf1*^±^ and *Nf1*^GFAP^ CKO mouse pups, however, the surface righting test was performed at PND14 in that study, which may be too late to find deviations in the developmental curve of these animals. Interestingly, our group has recently described a sexually dimorphic impact of the *Nf1*^±^ mutation in hippocampal neurochemistry and morphology, as well as a “camouflaging”-type behavior in female *Nf1*^±^ mice, during juvenile age [8]. Curiously, analysis of the data here presented regarding *Nf1*^±^ pups developmental tests shows that there is a consistent sexual dimorphism between WT^*Nf1*^ animals which is not recapitulated in transgenic animals; furthermore, in those same milestones tests, we found genotype-mediated differences specifically in males. Together, these results indicate the occurrence of neuronal mechanisms that dictate the emergence of developmental sex differences in NF1 mice. This is in line with previous clinical and animal research, which had identified biological sex as a prognostic factor for neuronal dysfunction in NF1 patients [28].

Concerning the *Tsc2*^±^ animal model, again, studies have looked into its early developmental milestones. Nevertheless, a thought-provoking study investigated the role of maternal genotype on the litter’s milestone development and found that although *Tsc2*^±^ pups had no significant alterations in early development, maternal heterozygosity was associated with developmental delay in WT^*Tsc2*^ pups [40]. On the other hand, several authors observed cognitive deficits [41, 42], social impairments [43, 44], and anxiety-related behaviors [45, 46] in the adult TSC2 animal model. Importantly, Saré et al. (2020) [47] reported the influence of sex in behavioral phenotypes of *Tsc2*^±^ mice, which is in accordance with findings in our group of sex-dependent behavioral deficits and region-specific sexual dimorphisms in cortical and hippocampal metabolites in juvenile *Tsc2*^±^ mice [7]. Together, these data highlight the great potential of the study of the *Tsc2*^±^ animal model in the context of neurodevelopmental disorders, with a focus on the effect of biological sex.

Finally, in this study, we performed a detailed analysis of the vocal repertoire of both mouse models. Here, we uncovered that significant sex- or genotype-related differences in USV number, and USV temporal and frequency modulation tend to be present at PND10 in *Tsc2*^±^ animals, while dimorphisms occur earlier in *Nf1*^±^ animals, in PND6 and 8 (Fig. 6). Maloney et al. (2018) [30] observed altered USV production from PND5 until PND9, with an increase in call production in *Nf1*^±^, which we report here specifically for male *Nf1*^±^ pups; however, the authors also report that *Nf1*^±^ mice presented lower mean frequency, which was not verified by our data, and that *Nf1*^*GFAP*^*-*CKO mouse pups demonstrated a decrease in USVs [30]. These partially conflicting results may be due to the different genetic backgrounds used in these studies. Further, the Greene-Colozzi et al. (2014) [40] study on the effect of maternal *Tsc2* heterozygosity found that all pups with a transgenic dam produced USVs in a reduced number or had a delay in reaching the peak number of calls; additionally, a delay in peak number of USVs was also observed in all mutant pups. This is in line with the data obtained in our study and brings to light the role of *Tsc2* heterozygosity in maternal-pup behavior, and its impact on neonatal USVs, which had already been hypothesized by Young et al. (2010) [37].

Other mouse models of ASD have been used to study USVs in neurodevelopmental disorders, describing communication deficits, namely, alterations in vocalization rates, number and duration of calls, frequency modulation, spectral properties, and temporal organization of USVs [48–52]. This data underscores the relevance of USVs as a robust biomarker for neurobehavioral development and maturation. Importantly, *Fmr1-*KO, *MALTT*, and *ProSAP1/Shank2*^*−/−*^ mouse models of ASD have been reported to emit aberrant USVs in infancy, as well as in juvenile and adult age [53–56], thus showing that communication deficits can be used as an early predictor for later phenotypes. The data here gathered indicates that different strains, and specifically, different mouse models of ASD, have distinct USV signatures and distinct maturation timelines of their vocal skills [57, 58]. In this sense, one should pay close attention to the timeframe and features analyzed in each study, to not miss relevant data, and to adequately formulate conclusions in the context of the specific developmental curve of each mouse model.

Additionally, in this work, across all analyzed USV features as well as in USV composition, we found a significant influence of biological sex, underscoring the potential to use both mouse models in the study of sexual dimorphic vocal biomarkers for early detection of ASD. Indeed, recently authors have shown sex-specific vocal phenotypes in *Fmr1-*KO and *Crmp-4-*KO mouse pups [56, 59, 60]. Interestingly, the contrary was reported in *Foxp1*-KO mice, as these lacked the sexual typical dimorphisms observed in WT pups, hinting at the role of the *Foxp1* gene in vocalization [61]. Finally, sex x gene/environment interactions leading to sex differences were found in spectral and temporal features of the USVs produced by *Mthfr*-KO and mice exposed to chlorpyrifos (CPF) during gestation [62]. Remarkably, postnatal exposure to CPF caused deficits in USV production in both male and female mice, with no sexual dimorphism [63], hinting that specifically prenatal, and not postnatal, environmental factors may affect key processes for proper vocal development in a sex-dependent manner.

## Conclusions

Our work has shown the strikingly different developmental profiles of two mouse models of ASD, with strong divergence regarding the effect of genotype and/or sex in the differences demonstrated by both mouse models in early development. Additionally, analysis of the vocal repertoire of each strain also revealed sex- and genotype-related differences, with clear distinctions between the two animal models, regarding the number of USVs, frequency modulation, temporal profile, and USV composition.

The main limitation of this study was the different genetic backgrounds of the two animal models used: *Tsc2*^±^ experimental group animals were generated from C57BL/N backcrossed to a C57BL/J, while *Nf1*^±^ animals had a hybrid background of C57BL/6N backcrossed to 129T2/SvEmsJ. As genetic background may influence ASD outcomes and manifestations, direct comparisons between the two animal models used here were not performed. Additionally, the body temperature of the pups was not measured during the USV recording protocol, which does not allow us to conclude regarding the potential interference of thermoregulatory mechanisms in the vocal performance of the animals [64]. However, the test facilities were always kept within an adequate, controlled ambient temperature range, so we believe that temperature effects were minimal in this study. Finally, future studies should aim to elucidate the biological underpinnings that lead to the observed altered vocalization signatures in the two ASD animal models. In fact, a deeper understanding of the disrupted neurophysiological mechanisms that result in aberrant USV production would strengthen the findings presented here.

This work broadens the current knowledge of the early developmental progression of two established mouse models of ASD. Future studies should further document phenotypical manifestations of the several existing animal models of autism, to build a comprehensive image of all the possible developmental profiles that could emerge from this very heterogeneous disorder, and thus work towards the design of more accurate therapies for affected individuals.

## Supplementary Information

Additional file 1. Developmental milestones tests. Representative images of the developmental milestones tests performed in this study, namely, surface righting (A), negative geotaxis (B), locomotion (C) and nest seeking (D) tests.

Additional file 2. Weight and length. Male and female mouse pups of *Nf1*^+/-^ and *Tsc2*^+/-^ experimental groups were weighted (A and C, respectively) and measured (B and D, respectively) throughout tested timepoints. Two-way ANOVA followed by Tukey’s multiple comparisons test. Data represented as mean ± SEM; n (male WT)= 18-19; n (female WT) = 19-20; n (male *Nf1*^+/-^) = 18-19; n (female *Nf1*^+/-^)= 20-22; n (male WT) = 16-18; n (female WT) = 16-20; n (male *Tsc2*^+/-^)= 16-19; n (female *Tsc2*^+/-^) = 25-30.

Additional file 3. Weight and Length of *Nf1*^+/-^ mouse model. Data represented as mean ± SEM. Two-way ANOVA followed by Tukey’s multiple comparisons test. Significant differences are marked as * (WT male vs mutant male), ^#^ (WT male vs WT female), ^+^ (mutant male vs mutant female) or ^$^ (WT female or mutant female).

Additional file 4. Weight and Length of *Tsc2*^+/-^ mouse model. Data represented as mean ± SEM. Two-way ANOVA followed by Tukey’s multiple comparisons test. Significant differences are marked as * (WT male vs mutant male), ^#^ (WT male vs WT female), ^+^ (mutant male vs mutant female) or ^$^ (WT female or mutant female).

Additional file 5. Developmental milestones of *Nf1*^+/-^ mouse model. Data represented as mean ± SEM. Two-way ANOVA (surface righting and negative geotaxis tests) or one-way ANOVA (locomotion and nest seeking tests), followed by Tukey’s multiple comparisons test. Significant differences are marked as * (WT male vs mutant male), ^#^ (WT male vs WT female), ^+^ (mutant male vs mutant female) or ^$^ (WT female or mutant female).

Additional file 6. Developmental milestones of *Tsc2*^+/-^ mouse model. Data represented as mean ± SEM. Two-way ANOVA (surface righting and negative geotaxis tests) or one-way ANOVA (locomotion and nest seeking tests), followed by Tukey’s multiple comparisons test. Significant differences are marked as * (WT male vs mutant male), ^#^ (WT male vs WT female), ^+^ (mutant male vs mutant female) or ^$^ (WT female or mutant female).

Additional file 7. Total number of USVs and USV duration of *Nf1*^+/-^ mouse model. Data represented as mean ± SEM. Two-way ANOVA followed by Tukey’s multiple comparisons test. Significant differences are marked as * (WT male vs mutant male), ^#^ (WT male vs WT female), ^+^ (mutant male vs mutant female) or ^$^ (WT female or mutant female).

Additional file 8. Total number of USVs and USV duration of *Tsc2*^+/-^ mouse model. Data represented as mean ± SEM. Two-way ANOVA followed by Tukey’s multiple comparisons test. Significant differences are marked as * (WT male vs mutant male), ^#^ (WT male vs WT female), ^+^ (mutant male vs mutant female) or ^$^ (WT female or mutant female).

Additional file 9. Total time emitting USVs, inter-USV interval and latency to produce first USV of *Nf1*^+/-^ mouse model. Data represented as mean ± SEM. Two-way ANOVA followed by Tukey’s multiple comparisons test. Significant differences are marked as * (WT male vs mutant male), ^#^ (WT male vs WT female), ^+^ (mutant male vs mutant female) or ^$^ (WT female or mutant female).

Additional file 10. Total time emitting USVs, inter-USV interval and latency to produce first USV of *Tsc2*^+/-^ mouse model. Data represented as mean ± SEM. Two-way ANOVA followed by Tukey’s multiple comparisons test. Significant differences are marked as * (WT male vs mutant male),^#^ (WT male vs WT female), ^+^ (mutant male vs mutant female) or ^$^ (WT female or mutant female).

Additional file 11. USV frequency and slope of *Nf1*^+/-^ mouse model. Data represented as mean ± SEM. Two-way ANOVA followed by Tukey’s multiple comparisons test. Significant differences are marked as * (WT male vs mutant male), ^#^ (WT male vs WT female), ^+^ (mutant male vs mutant female) or ^$^ (WT female or mutant female).

Additional file 12. USV frequency and slope of *Tsc2*^+/-^ mouse model. Data represented as mean ± SEM. Two-way ANOVA followed by Tukey’s multiple comparisons test. Significant differences are marked as * (WT male vs mutant male), ^#^ (WT male vs WT female), ^+^ (mutant male vs mutant female) or ^$^ (WT female or mutant female).

Additional file 13. USV syllable composition of *Nf1*^+/-^ mouse model. Data represented as mean ± SEM. Two-way ANOVA followed by Tukey’s multiple comparisons test. Significant differences are marked as * (WT male vs mutant male), ^#^ (WT male vs WT female), ^+^ (mutant male vs mutant female) or ^$^ (WT female or mutant female).

Additional File 14. USV syllable composition of *Tsc2*^+/-^ mouse model. Data represented as mean ± SEM. Two-way ANOVA followed by Tukey’s multiple comparisons test. Significant differences are marked as * (WT male vs mutant male), ^#^ (WT male vs WT female), ^+^ (mutant male vs mutant female) or ^$^ (WT female or mutant female).

## Data Availability

All data supporting the conclusions of this article are included within the article and its additional files. The datasets used and analyzed during the current study are available from the corresponding author on reasonable request.

## Abbreviations

ASD: Autism spectrum disorder
Nf1: Neurofibromatosis type 1
PND: Postnatal day
Tsc2: Tuberous sclerosis type 2
USV: Ultrasonic vocalizations
WT: Wild type
