# Supplementary figures and images for: Distinct early development trajectories in Nf1± and Tsc2± mouse models of autism
Source: J Neurodev Disord. 2025 Jul 26;17:42. doi: 10.1186/s11689-025-09624-6 (PMC12296589; doi:10.1186/s11689-025-09624-6)

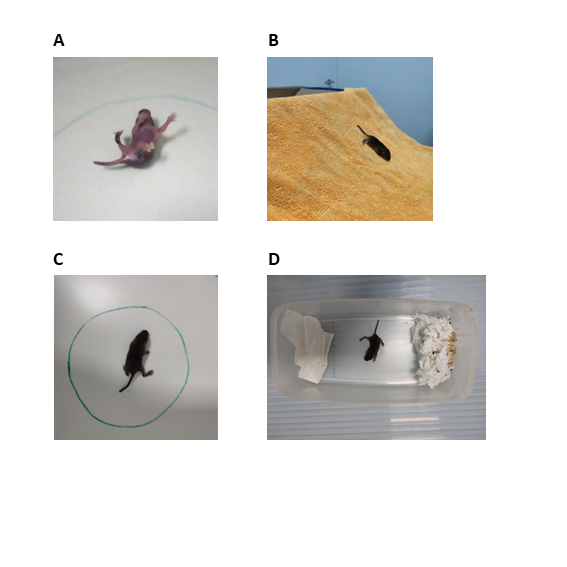

Supplement: Supplementary file 1 — Additional file 1. Developmental milestones tests. Representative images of the developmental milestones tests performed in this study, namely, surface righting (A), negative geotaxis (B), locomotion (C) and nest seeking (D) tests. [file 11689_2025_9624_MOESM1_ESM.tif]

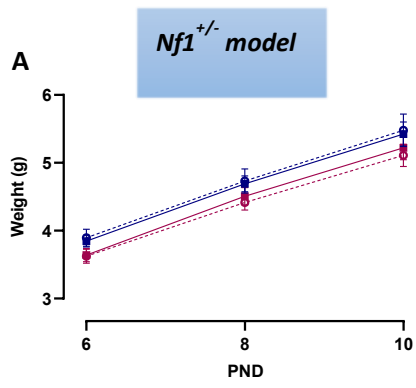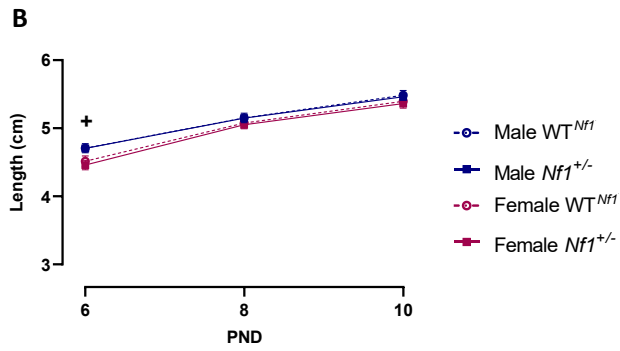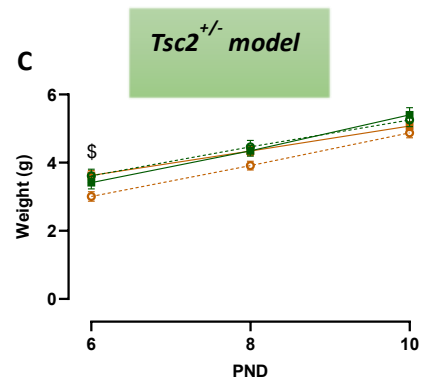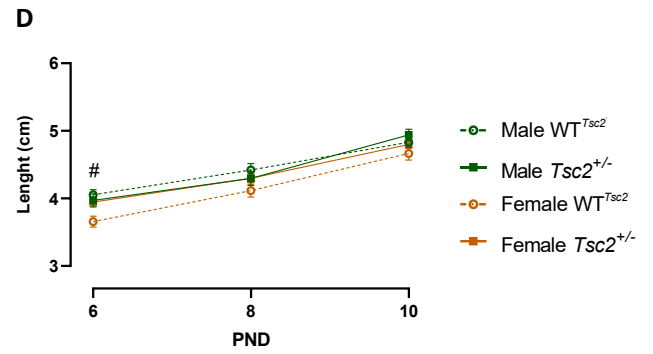

Supplement: Supplementary file 2 — Additional file 2. Weight and length. Male and female mouse pups of Nf1+/- and Tsc2+/- experimental groups were weighted (A and C, respectively) and measured (B and D, respectively) throughout tested timepoints. Two-way ANOVA followed by Tukey’s multiple comparisons test. Data represented as mean ± SEM; n (male WT)= 18-19; n (female WT) = 19-20; n (male Nf1+/-) = 18-19; n (female Nf1+/-)= 20-22; n (male WT) = 16-18; n (female WT) = 16-20; n (male Tsc2+/-)= 16-19; n (female Tsc2+/-) = 25-30. [file 11689_2025_9624_MOESM2_ESM.pdf]
