# Supplementary material for: Distinct early development trajectories in Nf1± and Tsc2± mouse models of autism
Source: J Neurodev Disord. 2025 Jul 26;17:42. doi: 10.1186/s11689-025-09624-6 (PMC12296589; doi:10.1186/s11689-025-09624-6)
Supplement: Supplementary file 3 — Additional file 3. Weight and Length of Nf1+/- mouse model. Data represented as mean ± SEM. Two-way ANOVA followed by Tukey’s multiple comparisons test. Significant differences are marked as * (WT male vs mutant male), # (WT male vs WT female), + (mutant male vs mutant female) or $ (WT female or mutant female). [file 11689_2025_9624_MOESM3_ESM.docx]

|  |  | PND6 | PND8 | PND10 |
| --- | --- | --- | --- | --- |
| Weight  mean±SEM (g) | Male WT*^Nf1^* | 3.89±0.125 | 4.73±0.179 | 5.48±0.239 |
|  | Male *Nf1*^+/-^ | 3.84±0.087 | 4.69±0.116 | 5.42±0.177 |
|  | Female WT*^Nf1^* | 3.62±0.104 | 4.42±0.111 | 5.11±0.165 |
|  | Female *Nf1*^+/-^ | 3.64±0.099 | 4.51±0.127 | 5.22±0.176 |
| Length  mean±SEM (cm) | Male WT*^Nf1^* | 4.70±0.067 | 5.15±0.070 | 5.48±0.070 |
|  | Male *Nf1*^+/-^ | 4.70±0.040 | 5.15±0.035 | 5.46±0.045 |
|  | Female WT*^Nf1^* | 4.52±0.078 | 5.08±0.046 | 5.40±0.049 |
|  | Female *Nf1*^+/-^ | **4.46±0.069^+^, p=0.0199** | 5.05±0.059 | 5.36±0.068 |
