# Supplementary material for: Distinct early development trajectories in Nf1± and Tsc2± mouse models of autism
Source: J Neurodev Disord. 2025 Jul 26;17:42. doi: 10.1186/s11689-025-09624-6 (PMC12296589; doi:10.1186/s11689-025-09624-6)
Supplement: Supplementary file 4 — Additional file 4. Weight and Length of Tsc2+/- mouse model. Data represented as mean ± SEM. Two-way ANOVA followed by Tukey’s multiple comparisons test. Significant differences are marked as * (WT male vs mutant male), # (WT male vs WT female), + (mutant male vs mutant female) or $ (WT female or mutant female). [file 11689_2025_9624_MOESM4_ESM.docx]

|  |  | PND6 | PND8 | PND10 |
| --- | --- | --- | --- | --- |
| Weight  mean±SEM (g) | Male WT*^Tsc2^* | 3.61±0.194 | 4.46±0.189 | 5.25±0.194 |
|  | Male *Tsc2*^+/-^ | 3.42±0.183 | 4.35±0.154 | 5.40±0.213 |
|  | Female WT*^Tsc2^* | 3.01±0.141 | 3.91±0.128 | 4.87±0.146 |
|  | Female *Tsc2*^+/-^ | **3.62±0.146^$^, p=0.0352** | 4.34±0.134 | 5.08±0.132 |
| Length  mean±SEM (cm) | Male WT*^Tsc2^* | 4.05±0.077 | 4.42±0.095 | 4.83±0.074 |
|  | Male *Tsc2*^+/-^ | 3.97±0.098 | 4.30±0.104 | 4.94±0.083 |
|  | Female WT*^Tsc2^* | **3.66±0.080^#^, p=0.0106** | 4.12±0.094 | 4.66±0.100 |
|  | Female *Tsc2*^+/-^ | 3.94±0.058 | 4.30±0.067 | 4.80±0.060 |
