# Supplementary material for: Distinct early development trajectories in Nf1± and Tsc2± mouse models of autism
Source: J Neurodev Disord. 2025 Jul 26;17:42. doi: 10.1186/s11689-025-09624-6 (PMC12296589; doi:10.1186/s11689-025-09624-6)
Supplement: Supplementary file 5 — Additional file 5. Developmental milestones of Nf1+/- mouse model. Data represented as mean ± SEM. Two-way ANOVA (surface righting and negative geotaxis tests) or one-way ANOVA (locomotion and nest seeking tests), followed by Tukey’s multiple comparisons test. Significant differences are marked as * (WT male vs mutant male), # (WT male vs WT female), + (mutant male vs mutant female) or $ (WT female or mutant female). [file 11689_2025_9624_MOESM5_ESM.docx]

| Developmental  Milestones |  | PND6 | PND8 | PND10 |
| --- | --- | --- | --- | --- |
| Surface Righting  mean±SEM (s) | Male WT*^Nf1^* | 3.08±0.311 | 3.15±0.588 |  |
|  | Male *Nf1*^+/-^ | **9.67±2.361**, p=0.0050** | 2.32±0.254 |  |
|  | Female WT*^Nf1^* | **8.35±2.464^#^, p=0.0419** | 2.46±0.356 |  |
|  | Female *Nf1*^+/-^ | 7.66±1.497 | 4.35±0.799 |  |
| Negative Geotaxis  mean±SEM (s) | Male WT*^Nf1^* | 17.38±3.053 | 20.83±2.130 |  |
|  | Male *Nf1*^+/-^ | 20.02±2.437 | **10.77±2.497*, p=0.0194** |  |
|  | Female WT*^Nf1^* | 18.28±3.442 | **10.11±1.247^#^, p=0.0133** |  |
|  | Female *Nf1*^+/-^ | 14.03±2.577 | 5.41±0.516 |  |
| Locomotion  mean±SEM (s) | Male WT*^Nf1^* |  | | 20.77±2.711 |
|  | Male *Nf1*^+/-^ |  |  | **29.56±0.442*, p=0.0189** |
|  | Female WT*^Nf1^* |  |  | **29.18±0.816^#^, p=0.0308** |
|  | Female *Nf1*^+/-^ |  |  | 24.87±2.702 |
| Nest Seeking  mean±SEM (s) | Male WT*^Nf1^* |  | | 25.71±7.466 |
|  | Male *Nf1*^+/-^ |  |  | 14.92±1.657 |
|  | Female WT*^Nf1^* |  |  | 16.78±4.704 |
|  | Female *Nf1*^+/-^ |  |  | 12.96±2.187 |
