# Supplementary material for: Distinct early development trajectories in Nf1± and Tsc2± mouse models of autism
Source: J Neurodev Disord. 2025 Jul 26;17:42. doi: 10.1186/s11689-025-09624-6 (PMC12296589; doi:10.1186/s11689-025-09624-6)
Supplement: Supplementary file 6 — Additional file 6. Developmental milestones of Tsc2+/- mouse model. Data represented as mean ± SEM. Two-way ANOVA (surface righting and negative geotaxis tests) or one-way ANOVA (locomotion and nest seeking tests), followed by Tukey’s multiple comparisons test. Significant differences are marked as * (WT male vs mutant male), # (WT male vs WT female), + (mutant male vs mutant female) or $ (WT female or mutant female). [file 11689_2025_9624_MOESM6_ESM.docx]

| Developmental  Milestones |  | PND6 | PND8 | PND10 |
| --- | --- | --- | --- | --- |
| Surface Righting  mean±SEM (s) | Male WT*^Tsc2^* | 11.46±3.252 | 2.06±0.376 |  |
|  | Male *Tsc2*^+/-^ | 13.19±3.131 | 1.75±0.166 |  |
|  | Female WT*^Tsc2^* | 17.94±2.886 | 2.76±0.504 |  |
|  | Female *Tsc2*^+/-^ | 14.50±2.287 | **10.40±2.106^+^, p=0.0263** |  |
| Negative Geotaxis  mean±SEM (s) | Male WT*^Tsc2^* | 23.29±2.342 | 17.14±2.596 |  |
|  | Male *Tsc2*^+/-^ | 21.01±2.478 | 19.38±2.637 |  |
|  | Female WT*^Tsc2^* | 23.95±2.007 | 15.67±2.487 |  |
|  | Female *Tsc2*^+/-^ | 24.60±1.710 | 19.33±1.891 |  |
| Locomotion  mean±SEM (s) | Male WT*^Tsc2^* |  | | 28.05±1.254 |
|  | Male *Tsc2*^+/-^ |  |  | 30.00±0.000 |
|  | Female WT*^Tsc2^* |  |  | 28.31±1.122 |
|  | Female *Tsc2*^+/-^ |  |  | **23.28±1.867^+^, p=0.0103** |
| Nest Seeking  mean±SEM (s) | Male WT*^Tsc2^* |  | | 46.71±9.530 |
|  | Male *Tsc2*^+/-^ |  |  | **16.10±3.488*, p=0.0336** |
|  | Female WT*^Tsc2^* |  |  | 51.98±9.668 |
|  | Female *Tsc2*^+/-^ |  |  | **25.65±4.601^$^, p=0.0384** |
