# Supplementary material for: Distinct early development trajectories in Nf1± and Tsc2± mouse models of autism
Source: J Neurodev Disord. 2025 Jul 26;17:42. doi: 10.1186/s11689-025-09624-6 (PMC12296589; doi:10.1186/s11689-025-09624-6)
Supplement: Supplementary file 7 — Additional file 7. Total number of USVs and USV duration of Nf1+/- mouse model. Data represented as mean ± SEM. Two-way ANOVA followed by Tukey’s multiple comparisons test. Significant differences are marked as * (WT male vs mutant male), # (WT male vs WT female), + (mutant male vs mutant female) or $ (WT female or mutant female). [file 11689_2025_9624_MOESM7_ESM.docx]

|  |  | PND6 | PND8 | PND10 |
| --- | --- | --- | --- | --- |
| Number of USVS  mean±SEM | Male WT | 515.4±24.1 | 379.5±40.6 | 372.7±20.9 |
|  | Male *Nf1*^+/-^ | 580.0±16.1 | **540.6±35.9**, p=0.0079** | 461.8±26.1 |
|  | Female WT | 505.5±28.3 | 465.0±37.1 | 381.6±59.5 |
|  | Female *Nf1*^+/-^ | **437.5±34.1^+^, p=0.0256** | 496.0±33.1 | 377.9±34.3 |
| USV duration  mean±SEM (s) | Male WT | 0.086±0.002 | 0.082±0.002 | 0.085±0.002 |
|  | Male *Nf1*^+/-^ | **0.078±0.002**, p=0.0089** | 0.081±0.002 | 0.083±0.001 |
|  | Female WT | 0.084±0.002 | 0.080±0.002 | 0.080±0.003 |
|  | Female *Nf1*^+/-^ | 0.083±0.002 | 0.079±0.002 | 0.081±0.002 |
