# Supplementary material for: Distinct early development trajectories in Nf1± and Tsc2± mouse models of autism
Source: J Neurodev Disord. 2025 Jul 26;17:42. doi: 10.1186/s11689-025-09624-6 (PMC12296589; doi:10.1186/s11689-025-09624-6)
Supplement: Supplementary file 8 — Additional file 8. Total number of USVs and USV duration of Tsc2+/- mouse model. Data represented as mean ± SEM. Two-way ANOVA followed by Tukey’s multiple comparisons test. Significant differences are marked as * (WT male vs mutant male), # (WT male vs WT female), + (mutant male vs mutant female) or $ (WT female or mutant female). [file 11689_2025_9624_MOESM8_ESM.docx]

|  |  | PND6 | PND8 | PND10 |
| --- | --- | --- | --- | --- |
| Number of USVS  mean±SEM | Male WT*^Tsc2^* | 371.4±35.5 | 302.9±30.1 | 255.5±21.6 |
|  | Male *Tsc2*^+/-^ | 332.1±34.8 | 334.0±28.0 | **135.5±26.6*, p=0.0177** |
|  | Female WT*^Tsc2^* | 334.1±22.5 | 338.0±19.7 | 271.0±24.0 |
|  | Female *Tsc2*^+/-^ | 319.6±31.3 | 293.2±26.4 | **116.2±13.1^$$$^, p=0.0002** |
| USV duration  mean±SEM (s) | Male WT*^Tsc2^* | 0.024±0.002 | 0.020±0.001 | 0.019±0.002 |
|  | Male *Tsc2*^+/-^ | 0.027±0.002 | 0.022±0.002 | 0.021±0.002 |
|  | Female WT*^Tsc2^* | 0.026±0.003 | 0.023±0.002 | 0.020±0.001 |
|  | Female *Tsc2*^+/-^ | 0.025±0.002 | 0.020±0.001 | **0.013±0.001,**  **^+^ p=0.0373, ^$^p=0.0441** |
