# Supplementary material for: Distinct early development trajectories in Nf1± and Tsc2± mouse models of autism
Source: J Neurodev Disord. 2025 Jul 26;17:42. doi: 10.1186/s11689-025-09624-6 (PMC12296589; doi:10.1186/s11689-025-09624-6)
Supplement: Supplementary file 9 — Additional file 9. Total time emitting USVs, inter-USV interval and latency to produce first USV of Nf1+/- mouse model. Data represented as mean ± SEM. Two-way ANOVA followed by Tukey’s multiple comparisons test. Significant differences are marked as * (WT male vs mutant male), # (WT male vs WT female), + (mutant male vs mutant female) or $ (WT female or mutant female). [file 11689_2025_9624_MOESM9_ESM.docx]

|  |  | PND6 | PND8 | PND10 |
| --- | --- | --- | --- | --- |
| Total USV time  mean±SEM (s) | Male WT*^Nf1^* | 39.87±2.538 | 38.20±3.557 | 33.25±2.337 |
|  | Male *Nf1*^+/-^ | 38.97±2.889 | 36.57±3.579 | 33.87±2.761 |
|  | Female WT*^Nf1^* | 38.39±3.351 | 38.05±3.518 | 31.83±4.802 |
|  | Female *Nf1*^+/-^ | 37.06±3.278 | 37.83±2.662 | 32.20±2.716 |
| Inter-USV interval  mean±SEM (s) | Male WT*^Nf1^* | 0.50±0.028 | 0.62±0.076 | 0.73±0.051 |
|  | Male *Nf1*^+/-^ | 0.48±0.032 | 0.65±0.080 | 0.61±0.035 |
|  | Female WT*^Nf1^* | 0.53±0.040 | 0.58±0.051 | **1.87±0.607^####^, p<0.0001** |
|  | Female *Nf1*^+/-^ | 0.55±0.034 | 0.58±0.046 | **0.73±0.082^$$$$^, p<0.0001** |
| Latency to first call  mean±SEM (s) | Male WT*^Nf1^* | 3.98±1.330 | 0.72±0.343 | 1.04±0.572 |
|  | Male *Nf1*^+/-^ | 3.51±0.554 | 1.56±0.673 | 2.26±0.746 |
|  | Female WT*^Nf1^* | 3.19±0.258 | 1.10±0.537 | **3.89±0.728^#^, p=0.0266** |
|  | Female *Nf1*^+/-^ | **0.69±0.268, ^+^p=0.0206, ^$^p=0.0450** | 2.83±0.913 | 1.80±0.637 |
