# Supplementary material for: Distinct early development trajectories in Nf1± and Tsc2± mouse models of autism
Source: J Neurodev Disord. 2025 Jul 26;17:42. doi: 10.1186/s11689-025-09624-6 (PMC12296589; doi:10.1186/s11689-025-09624-6)
Supplement: Supplementary file 10 — Additional file 10. Total time emitting USVs, inter-USV interval and latency to produce first USV of Tsc2+/- mouse model. Data represented as mean ± SEM. Two-way ANOVA followed by Tukey’s multiple comparisons test. Significant differences are marked as * (WT male vs mutant male),# (WT male vs WT female), + (mutant male vs mutant female) or $ (WT female or mutant female). [file 11689_2025_9624_MOESM10_ESM.docx]

|  |  | PND6 | PND8 | PND10 |
| --- | --- | --- | --- | --- |
| Total USV time  mean±SEM (s) | Male WT*^Tsc2^* | 9.34±1.198 | 6.66±1.214 | 7.13±1.218 |
|  | Male *Tsc2*^+/-^ | 9.99±1.398 | 7.84±1.197 | **2.67±0.348*, p=0.0409** |
|  | Female WT*^Tsc2^* | 9.20±1.283 | 9.94±1.140 | 6.14±0.688 |
|  | Female *Tsc2*^+/-^ | 7.23±0.959 | 7.42±0.985 | 2.45±0.419 |
| Intercall interval  mean±SEM (s) | Male WT*^Tsc2^* | 1.00±0.165 | 1.07±0.130 | 1.49±0.250 |
|  | Male *Tsc2*^+/-^ | 0.96±0.142 | 0.95±0.118 | **2.50±0.364*, p=0.0368** |
|  | Female WT*^Tsc2^* | 1.19±0.229 | 0.82±0.062 | 1.21±0.152 |
|  | Female *Tsc2*^+/-^ | 1.11±0.151 | 1.48±0.198 | **2.66±0.421^$$$$^, p<0.0001** |
| Latency to first call  mean±SEM (s) | Male WT*^Tsc2^* | 2.91±0.899 | 13.91±5.542 | 14.87±3.432 |
|  | Male *Tsc2*^+/-^ | 3.05±1.282 | 2.67±0.601 | 14.18±5.176 |
|  | Female WT*^Tsc2^* | 10.73±2.796 | 3.57±1.224 | **3.24±0.891^#^, p=0.0468** |
|  | Female *Tsc2*^+/-^ | 6.45±1.875 | 3.49±0.812 | **25.57±7.572^$$$$^, p<0.0001** |
