# Supplementary material for: Distinct early development trajectories in Nf1± and Tsc2± mouse models of autism
Source: J Neurodev Disord. 2025 Jul 26;17:42. doi: 10.1186/s11689-025-09624-6 (PMC12296589; doi:10.1186/s11689-025-09624-6)
Supplement: Supplementary file 11 — Additional file 11. USV frequency and slope of Nf1+/- mouse model. Data represented as mean ± SEM. Two-way ANOVA followed by Tukey’s multiple comparisons test. Significant differences are marked as * (WT male vs mutant male), # (WT male vs WT female), + (mutant male vs mutant female) or $ (WT female or mutant female). [file 11689_2025_9624_MOESM11_ESM.docx]

|  |  | PND6 | PND8 | PND10 |
| --- | --- | --- | --- | --- |
| Mean frequency  mean±SEM (kHz) | Male WT*^Nf1^* | 63.31±0.726 | 62.21±0.875 | 65.08±0.778 |
|  | Male *Nf1*^+/-^ | 65.06±1.754 | 66.06±1.694 | 65.08±0.783 |
|  | Female WT*^Nf1^* | 64.79±1.848 | 67.31±1.342 | 65.67±0.473 |
|  | Female *Nf1*^+/-^ | 63.77±1.071 | 66.43±1.976 | 65.59±1.392 |
| Slope  mean±SEM (kHz/s) | Male WT*^Nf1^* | -26.09±8.325 | -24.70±4.542 | 3.157±4.946 |
|  | Male *Nf1*^+/-^ | -27.34±6.031 | -26.72±3.865 | -3.70±3.483 |
|  | Female WT*^Nf1^* | 30.69±2.764 | -29.58±3.288 | -6.26±6.559 |
|  | Female *Nf1*^+/-^ | -35.27±4.461 | -26.94±4.560 | -18.17±5.052 |
