# Supplementary material for: Distinct early development trajectories in Nf1± and Tsc2± mouse models of autism
Source: J Neurodev Disord. 2025 Jul 26;17:42. doi: 10.1186/s11689-025-09624-6 (PMC12296589; doi:10.1186/s11689-025-09624-6)
Supplement: Supplementary file 12 — Additional file 12. USV frequency and slope of Tsc2+/- mouse model. Data represented as mean ± SEM. Two-way ANOVA followed by Tukey’s multiple comparisons test. Significant differences are marked as * (WT male vs mutant male), # (WT male vs WT female), + (mutant male vs mutant female) or $ (WT female or mutant female). [file 11689_2025_9624_MOESM12_ESM.docx]

|  |  | PND6 | PND8 | PND10 |
| --- | --- | --- | --- | --- |
| Mean frequency  mean±SEM (kHz) | Male WT*^Tsc2^* | 74.69±2.288 | 80.56±1.365 | 80.21±1.324 |
|  | Male *Tsc2*^+/-^ | 73.90±2.246 | 79.20±1.177 | 80.82±0.823 |
|  | Female WT*^Tsc2^* | 74.54±1.733 | 75.89±1.321 | 79.52±1.100 |
|  | Female *Tsc2*^+/-^ | **80.34±1.237, ^++^p=0.0061, ^$^ p=0.0127** | **81.17±0.959^$^, p=0.0321** | 80.75±0.992 |
| Slope  mean±SEM (kHz/s) | Male WT*^Tsc2^* | -127.5±27.72 | -216.4±30.81 | -208.8±33.44 |
|  | Male *Tsc2*^+/-^ | -202.6±27.90 | -244.7±27.12 | -215.5±37.93 |
|  | Female WT*^Tsc2^* | -138.9±24.73 | -154.4±33.89 | -129.3±41.99 |
|  | Female *Tsc2*^+/-^ | -204.9±27.25 | **-273.2±33.10^$^, p=0.0292** | **-278.8±31.67^$$^, p=0.0044** |
