# Supplementary material for: Distinct early development trajectories in Nf1± and Tsc2± mouse models of autism
Source: J Neurodev Disord. 2025 Jul 26;17:42. doi: 10.1186/s11689-025-09624-6 (PMC12296589; doi:10.1186/s11689-025-09624-6)
Supplement: Supplementary file 13 — Additional file 13. USV syllable composition of Nf1+/- mouse model. Data represented as mean ± SEM. Two-way ANOVA followed by Tukey’s multiple comparisons test. Significant differences are marked as * (WT male vs mutant male), # (WT male vs WT female), + (mutant male vs mutant female) or $ (WT female or mutant female). [file 11689_2025_9624_MOESM13_ESM.docx]

|  |  | PND6 | PND8 | PND10 |
| --- | --- | --- | --- | --- |
| Single USVs  (% of total USVs) | Male WT*^Nf1^* | 53.89±6.90 | 42.06±4.13 | 33.51±2.45 |
|  | Male *Nf1*^+/-^ | 50.91±8.22 | 47.23±7.42 | 37.53±2.25 |
|  | Female WT*^Nf1^* | 48.78±4.54 | 43.06±3.93 | 33.27±2.19 |
|  | Female *Nf1*^+/-^ | 46.89±3.98 | 43.66±3.89 | 41.78±2.21 |
| Multisyllabic USVs  (% of total USVs) | Male WT*^Nf1^* | 10.45±1.52 | 29.11±4.88 | 32.76±3.46 |
|  | Male *Nf1*^+/-^ | **24.54±1.19**, p=0.0076** | 19.82±3.52 | 37.29±2.04 |
|  | Female WT*^Nf1^* | **22.79±4.75^#^, p=0.0410** | 32.81±3.35 | 36.00±1.89 |
|  | Female *Nf1*^+/-^ | 27.48±3.65 | **17.32±1.29^$^, p=0.0175** | **27.33±2.40^+^, p=0.0350** |
| Stacked USVs  (% of total USVs) | Male WT*^Nf1^* | 30.62±7.14 | 22.96±6.64 | 36.36±5.83 |
|  | Male *Nf1*^+/-^ | 32.95±6.96 | 32.36±5.03 | 27.69±2.81 |
|  | Female WT*^Nf1^* | 31.26±5.48 | 23.94±4.17 | 30.58±3.26 |
|  | Female *Nf1*^+/-^ | 32.07±4.81 | 36.87±4.75 | 30.71±2.77 |
