# Supplementary material for: Distinct early development trajectories in Nf1± and Tsc2± mouse models of autism
Source: J Neurodev Disord. 2025 Jul 26;17:42. doi: 10.1186/s11689-025-09624-6 (PMC12296589; doi:10.1186/s11689-025-09624-6)
Supplement: Supplementary file 14 — Additional File 14. USV syllable composition of Tsc2+/- mouse model. Data represented as mean ± SEM. Two-way ANOVA followed by Tukey’s multiple comparisons test. Significant differences are marked as * (WT male vs mutant male), # (WT male vs WT female), + (mutant male vs mutant female) or $ (WT female or mutant female). [file 11689_2025_9624_MOESM14_ESM.docx]

|  |  | PND6 | PND8 | PND10 |
| --- | --- | --- | --- | --- |
| Single USVs  (% of total USVs) | Male WT*^Tsc2^* | 45.93±4.863 | 57.11±3.994 | 60.90±3.030 |
|  | Male *Tsc2*^+/-^ | 41.76±5.273 | 50.18±4.180 | 64.32±4.908 |
|  | Female WT*^Tsc2^* | 38.76±3.174 | **38.87±3.743^##^, p=0.0095** | 55.48±3.533 |
|  | Female *Tsc2*^+/-^ | 49.23±3.488 | **53.22±3.267^$^, p=0.0308** | 63.02±3.233 |
| Multisyllabic USVs  (% of total USVs) | Male WT*^Tsc2^* | 34.87±4.306 | 20.95±3.787 | 13.89±1.945 |
|  | Male *Tsc2*^+/-^ | **18.33±2.657*, p=0.0185** | 20.60±3.513 | 7.73±1.645 |
|  | Female WT*^Tsc2^* | 37.88±4.166 | 28.75±4.564 | 12.37±2.007 |
|  | Female *Tsc2*^+/-^ | **24.62±2.785^$^, p=0.0250** | 25.27±3.035 | 10.48±1.139 |
| Stacked USVs  (% of total USVs) | Male WT*^Tsc2^* | 21.60±4.204 | 21.94±2.726 | 25.21±3.601 |
|  | Male *Tsc2*^+/-^ | **41.63±5.133**, p=0.0038** | 29.22±3.682 | 27.95±4.392 |
|  | Female WT*^Tsc2^* | 21.87±2.561 | 29.90±3.856 | 32.15±3.075 |
|  | Female *Tsc2*^+/-^ | **26.15±2.917^+^, p=0.0164** | 24.50±3.452 | 26.50±2.996 |
